# The Foreign Relations Committee

**Published:** 1899-03

**Authors:** 


### THE FOREIGN RELATIONS COMMITTEE.

Upon another page will be found a partial report of the work of this committee. It comprises conclusions at which it arrived at its recent meeting at Cincinnati in regard to the Foreign Advisory Committee, decided upon at the last meeting of the National Association of Dental Faculties. It will be observed that this foreign committee has been only partially completed. It is presumed that the parties to fill the vacancies will be selected on or before the meeting of the Faculties at Niagara Falls in August.

The most interesting part of the work is outlined in the letters accompanying this. The energetic work of Dr. Barrett, chairman, has aroused the educational body in Illinois as never before. The press of Chicago has taken up this matter, and has denounced the whole business in no measured terms. The result must be that the Illinois Legislature will be forced, by outraged public opinion, to enact a law that will drive these fraudulent diploma manufactories from the State. The question is, What will follow when this is accomplished? Will these fraudulent dealers turn up elsewhere? This will depend upon the laxity of State laws, but it is presumed they will find none so favorable for their purpose as the constitution of the State of Illinois.

The dental profession and all educational institutions owe a debt of gratitude to Dr. Barrett for his work in unearthing these establishments in Chicago, for while efforts in this direction had been made previously, they seemed to amount to but little in arousing to active work. Much of this is due President Harper, of the Chicago University, who has entered into this labor with characteristic energy, and through his great influence has aided in rousing public opinion to an extent that, probably, without this might not have been accomplished so speedily.

From a private letter received from Dr. Barrett the following quotations are made. They forcibly give his views upon the subject.

"I wish to give you some additional facts.

"*First.* We have begun suit against about the worst of the fraud colleges. This we shall push as fast as possible. We think we have the necessary testimony.

"*Second.* We shall, we think, get the necessary legislation for the repeal of the bad Illinois law.

"*Third.* The committee of the Universities of Illinois has had a bill introduced in the Legislature of Illinois, but it is so drastic in its provisions that it bids fair to fail. Another that will be drafted after my ideas, and which will go no farther than it can without material opposition, will in due time take its place and will go through.

"*Fourth.* What we accomplish must be mainly done through legislation. The fraud college that we have commenced proceedings against has *eight* separate charters, each under a separate name. Of course, if beaten on one it will bring out another. You may say, then, What is the use in beginning suits when legislation alone can accomplish the end by reason of the repeal of the law under which these charters were issued? I answer that, (1) we must keep up the agitation. If nothing is being done to intimidate the work of issuing charters, this will still go on. We think we can stop that for the present. (2) If we do get legislation these suits will be far enough advanced to get some of the men in prison, we hope. We shall not try to get a decision on them until the outcome of the efforts for legislation is reached.

"*Fifth.* I have said that the college that we have begun action against has eight different charters. It has openly offered for sale thirty-six different degrees, in Literature, in the Arts, in Science, in Medicine, in Dentistry, in Law, in Pharmacy, in Veterinary Medicine, in Music, and in Theology. In fact, they will manufacture and turn over to any one, while he waits, any known degree.

"The first open intimation of the existence of this state of affairs was given in my report at Omaha. You will see by some of the papers that this report awakened President Harper, and he used it in a circular issued to the presidents of Illinois and Western colleges and universities, read it in a public meeting, and sent it out broadcast and to members of the Legislature, or extracts from it."

The amount of labor given to this matter by Dr. Barrett can hardly be appreciated by those unfamiliar with such work, but the success of his efforts thus far deserves, and we feel assured they will receive, the earnest thanks of every well-wisher for educational purity and progress.

